# Special Analytical and Health Commission on Tinned and Preserved Food

**Published:** 1906-07-21

**Authors:** 


### SPECIAL ANALYTICAL AND HEALTH COMMISSION ON TINNED AND PRESERVED FOOD.

### IV. PRESERVED FOODS CONSISTING LARGELY OF FAT.

While meat or proteid food must be regarded as the dietetic element essentially utilised in keeping the machinery of the human organism in repair or as the tissue builder and sustainer, fats and carbohydrates are the constituents of our diet which provide us with the actual energy for moving, breathing, and working. To understand the functions of diet rightly one may perhaps best regard the human organism as a machine for converting the potential chemical energy of foodstuffs into the kinetic energy of life. The essential chemical change which is the foundation of the power the body possesses of converting potential into kinetic energy is oxidation. We ingest complicated chemical substances possessing, somewhat like nitro-glycerine or gunpowder, a high potential energy, and we oxidise them, and from the resulting explosion—for explosion it essentially is—there remain simply the energy which is spent in living processes, the temperature at which our body is maintained, and what we may term a chemical rest, consisting of carbon dioxide, water, and urea. The kind of biological energy which is developed from a given kind of food depends almost entirely upon the individual. Let us contemplate for a moment one of the automatic delivery machines which many of us use frequently at railway stations, and consider that the pennies are always the same, but according to the slot they are put in will produce cigarettes or chocolates. From the same meal a poet will produce a sonnet, and a sulky girl the sulks; and having produced them, both will be hungry. The liquid assets, to use a financial term, of the human machine are the fats and carbohydrates of its food. We shall consider in a subsequent article the question of carbohydrates in connection with the all-important problem of food preservation. This will chiefly be dealt with when we come to consider fruit and vegetable preserves and confectionery, and, flippant as it may sound, jam *versus* dripping is one of the dietetic problems of the day. In the present article we shall confine our attention to fats and those animal foods which supply them.

For the sake of convenience, although not strictly accurately, we classify food according to the number of so-called calories a given weight of it will produce. The calorie is the amount of heat required to raise one litre of water 1° C. To measure the calorimetric value, or the energy value as we may term it, of any kind of food, one simply observes how many degrees Centigrade one litre of water can be raised by the complete oxidation of one gramme of it. It is estimated that an individual of average weight requires approximately 3,000 calories, or energy units, to perform an ordinary day's work; and to suit the convenience of the human machine, of these 3,000 calories at least 500 should be provided by fat. Fat has the highest calorimetric or energy value of any of the constituents of our diet. It is in this respect, weight for weight, more than twice as valuable as proteid (meat) or carbohydrate (starch and sugar). Hence, from a given bulk of fat more energy-value is obtained than from any other food. Although some kinds of fat are expensive, there is a mass of animal fat which can be obtained, when we take into consideration its food value, at a very cheap rate, such, for instance, as oleo-margarine. Unfortunately, the human organism can only assimilate a certain quantity of fat per diem without experiencing some digestive difficulty. The average person can deal with from 2½ oz. of fat in the summer to 3½oz. of fat in the winter per diem. The inhabitants of cold countries obtain much more of the necessary carbon of their diet in the form of fat than the inhabitants of warm countries, and it is interesting in this connection to compare the diet of an Esquimo with that of an inhabitant of the Nile Valley. The explanation of this is probably as much ethnographical as physiological; butter (the typical fat of our diet), for instance, being eagerly consumed and considered a luxury in hot countries. In this connection it will doubtless be remembered that one of the many attractions with which Jael enticed the weary Sisera was butter in a lordly dish, and that in Isaiah butter and honey are described as the diet of Immanuel, that he may know how to refuse the evil and choose the good.

The chief fats of an ordinary diet are of three kinds—namely, fats derived from the vegetable kingdom, such, for instance, as the fixed oils, butter, and fats derived from the animal kingdom. It is to these latter that our attention is specially directed in this article. The digestibility of fat depends to a great extent upon its flavour and its melting point. Certain fats, as, for instance, the fat in certain kinds of birds and fishes, invariably cause digestive troubles; further, beef and hog fat are more digestible than mutton fat, which latter has a higher melting point. The actual mechanical condition of a fat when ingested also has a powerful influence upon its digestibility, and we are capable of assimilating many fats in a fine state of mechanical division which we could not assimilate in lumps.

### Bacon.

Bacon consists essentially of fat. The average composition of good bacon should be as follows: Water, 9 per cent.; nitrogenous matter or proteid, 9 per cent.; fat, 75.75 per cent.; ash or mineral matter, 5.38 per cent. Bacon is made from different parts of the hog, and may be smoked or non-smoked; in any case it is what is called cured. Curing is a species of pickling, the flesh being treated with a brine consisting of salt, saltpetre, and very often, if the bacon is required to be mild cured, of some boron preservative. The effect of curing is essentially to preserve and to withdraw water; hence bacon must be regarded as a concentrated or relatively water-free food. Ordinary beef, for instance, contains from 60 per cent. to 70 per cent. of water, bacon containing about 10 per cent. The saltpetre has two functions: it acts as a preservative, and, in addition to this, the decolouration of the flesh which always takes place when meat is dried, owing to the decomposition of the hæmoglobin into meth-hæmoglobin and hæmatin, is counteracted by this substance, and flesh cured by a pickle containing it remains of a pleasant red colour.

We have received two samples of bacon, one from Messrs. Oake, Woods and Co. This bacon was of excellent quality, well up to the standard of what bacon ought to be, and of an exceedingly pleasant flavour. We received another sample of bacon of a very unique and useful character. We refer to the canned, sliced, and cooked breakfast bacon of Hunter's Handy Ham Co., London and Liverpool. This is also excellent bacon, and is quite ready for use. Fat, as we take it in bacon, seems to be on account of its mechanical form, and probably also on account of its physical constitution, relatively easy of digestion. It can often be taken in large quantities by persons who are intolerant to other forms of fat. It is invaluable in certain wasting diseases—*e.g.* phthisis, and also in the feeding of invalid children. In that we can to a large extent replace the carbohydrates of the diet by fat, bacon is also of great service in the feeding of diabetics.

### Lard and Suet.

Messrs. Oake, Woods and Co. have submitted a sample of their lard. Lard is the purified fat of the hog. The leafy fatty masses of the omentum and perinephritic tissues technically known as flare are first well washed and dried. They are then pounded in a mortar to release the fat from its membranous envelopes. The resulting mass is gently heated to a temperature not exceeding 57° C. in a water-bath. It is then strained carefully to entirely free it from membranous material. It is allowed slowly to cool, being stirred the while to prevent the crystallisation of palmitine and stearin, which if it occurred, would render the lard granular. No air must be introduced during this process, or the lard is apt to become rancid. Good lard should be soft, white, and of uniform consistency, melting at about 37° C. and forming a perfectly clear liquid at somewhat higher temperature. It should have a slightly fatty odour, should be neutral to litmus, and be entirely soluble in ether. It consists chemically of approximately 60 per cent. of olein, the remainder being made up of about equal parts of palmitine and stearin. Rancidity may develop upon keeping lard, and is generally due to hydrolysis, and is especially apt to occur upon exposure to strong light, hence lard should be kept in the dark. The sample submitted by Messrs. Oake, Woods and Co. is in every way a pure and satisfactory lard, and is evidently made carefully, and keeps well.

### Suet.

We have received two kinds of beef suet from Messrs. Hugon and Co., of Manchester—namely, shredded beef suet, Atora brand, and refined beef suet, Atora brand. Suet is the name given to the masses of fat occurring in the omentum and sub-abdominal tissues of the ox and the sheep, and these two kinds are named beef and mutton suet respectively. The fat is, under natural conditions, contained in a connective tissue meshwork, this meshwork being so completely enveloping that if you take suet as it is and treat it with a strong fat solvent, such, for instance, as ether, the connective tissue prevents the ether from getting at the fat and dissolving it out. What ether cannot do the human digestive juices cannot do, and raw suet, as it exists naturally, is indigestible, and will only yield a very small quantity of fat for the purposes of assimilation. This quality of suet explains the necessity, when ordinary suet is used, of finely mincing it, this process breaking down mechanically the connective tissue cell-walls and liberating the fat. This, of course, can be done on the manufacturing scale, and the result which may well be termed shredded suet is most convenient for domestic purposes. Messrs. Hugon's shredded suet, for instance, is supplied in fine mechanical division, and lends itself excellently to culinary purposes. We can, if we wish, proceed further with regard to the preparation of suet for dietetic purposes; we can not only break it up mechanically, but we can treat the result carefully by heat and filtration, and thus get rid entirely of the membranous matter. Such an article is generally termed prepared suet, and a type of this class is the *sevum preparatum* of the British Pharmacopœia. As a matter of fact the Pharmacopœia uses mutton suet, because this melts at a higher temperature than beef suet, and hence, as its use is chiefly as a basis for ointments, an advantage is obtained. Messrs. Hugon and Co. have also sent us a sample of such a prepared suet which we regard as of good quality. The characteristic of prepared suets is that they all possess a higher melting point than their respective natural suets, and for this reason are probably not quite so digestible; they are specially adapted for use instead of butter or oil for frying purposes. Suet is an important substance from the nutritional standpoint, as the following analyses of beef and mutton suets will show. The figures for good beef suet are: Water, 9.67 per cent.; nitrogenous substances (membrane), 1.16 per cent.; fat, 88.88 per cent. The average composition of mutton suet is: Water, 10.48 per cent.; membrane, 1.66 per cent.; fat, 89.88 per cent. Beef suet melts at 37° C.-38° C., mutton suet at 38° C.-40° C. If either suet, after being mechanically treated as described above, is heated to 50° C., the fat constituents melt and float and the membranous portion sinks; they can then be separated, as is done in the manufacture of prepared suet, and the residue can be utilised as a cattle food.

